# A class of derivative-free trust-region methods with interior backtracking technique for nonlinear optimization problems subject to linear inequality constraints

**DOI:** 10.1186/s13660-018-1698-7

**Published:** 2018-05-09

**Authors:** Jing Gao, Jian Cao

**Affiliations:** 1 School of Mathematics and Statistics, Beihua University, Jilin, P.R. China; 2 School of Information Technology and Media, Beihua University, Jilin, P.R. China

**Keywords:** 49M37, 65K05, 90C30, 90C51, Affine scaling, Trust-region method, Inequality constraints, Derivative-free optimization, Interior backtracking technique

## Abstract

This paper focuses on a class of nonlinear optimization subject to linear inequality constraints with unavailable-derivative objective functions. We propose a derivative-free trust-region methods with interior backtracking technique for this optimization. The proposed algorithm has four properties. Firstly, the derivative-free strategy is applied to reduce the algorithm’s requirement for first- or second-order derivatives information. Secondly, an interior backtracking technique ensures not only to reduce the number of iterations for solving trust-region subproblem but also the global convergence to standard stationary points. Thirdly, the local convergence rate is analyzed under some reasonable assumptions. Finally, numerical experiments demonstrate that the new algorithm is effective.

## Introduction

In this paper, we analyze the solution of following nonlinear optimization problem:

1 $$ \begin{gathered} \min\quad f(x) \\ \text{s.t.}\quad Ax\geqslant b, \end{gathered} $$

where $f(x)$ is a nonlinear twice continuously differentiable function, but its first-order or second-order derivatives are not explicitly available, $A\stackrel{\mathrm{def}}{=}[a_{1}^{T},a_{2}^{T},\ldots,a_{m}^{T}]^{T} \in\Re^{m\times n}$ with $a_{i}^{T} \in\Re^{n}$ and $b\stackrel{\mathrm{def}}{=}[b_{1},b_{2},\ldots,b_{m}]^{T} \in\Re ^{m}$. The feasible set, in (1), is denoted by $\Omega\stackrel{\mathrm{def}}{=} \{ x\in\Re^{n}| Ax\geqslant b \} $ and the strict interior feasible set is $\operatorname {int}(\Omega)\stackrel{\mathrm{def}}{=} \{ x\in\Re^{n}| Ax> b \} $.

### Affine-scaling matrix for inequality constraints

The KKT system of (1) is

2 $$\begin{aligned}& \nabla f(x)-A^{T}\lambda_{f}=0, \\& \operatorname {diag}\{Ax-b\}\lambda_{f}=0, \\& Ax-b\geqslant0,\quad\quad \lambda_{f}\geqslant 0, \end{aligned}$$

where $\lambda_{f}\in\Re^{m}$. A feasibility $x^{*}$ is said to be the stationary point for problem (1), if there exists a vector $0\leqslant\lambda_{f^{*}}\in\Re^{m}$ such that the KKT system (2) holds.

To solve this KKT system, some effective affine-scaling algorithms are designed. Reference [1] proposed an affine-scaling trust-region method with interior-point technique for bound-constrained semismooth equations. Reference [2] introduced affine-scaling interior-point Newton methods for bound-constrained nonlinear optimization. In particular, [3] proved the superlinear and quadratic convergence properties of affine-scaling interior-point Newton methods for bound optimization problems without strict complementarity assumption. Different affine-scaling matrix denotes different algorithm. In [4], the Dikin affine scaling was denoted by

3 $$ D(x)\stackrel{\mathrm{def}}{=}\operatorname {diag}\{Ax-b\} \quad \mbox{and} \quad D_{k}\stackrel{\mathrm{def}}{=}D(x_{k}). $$

Moreover, diagonal matrix $C_{f_{k}}\stackrel{\mathrm{def}}{=} \operatorname {diag}\{ \vert \lambda_{f_{k}} \vert \}$ was presented in [4]. Then $\lambda_{f_{k}}$ could be obtained as a least-squares Lagrangian multiplier approximation computed by

4 $$ \left [ \begin{matrix} A^{T} \\ -D_{k}^{\frac{1}{2}} \end{matrix} \right ] \lambda_{f_{k}}\stackrel{\mathrm{L.S.}}{=} \left [ \begin{matrix} \nabla f_{k} \\ 0 \end{matrix} \right ] . $$

One efficient affine-scaling interior-point trust-region model is the one which is presented in [5] and [6], written in the form

5 $$\begin{aligned} \begin{gathered} \min\quad q_{f_{k}}(p)=\nabla f_{k}^{T}p+ \frac{1}{2}p^{T}H_{f_{k}} p+\frac{1}{2}p^{T}A^{T}D_{k}^{-1}C_{f_{k}}Ap \\ \mbox{subject to}\quad \bigl\Vert \bigl[p;D_{k}^{-{\frac{1}{2}}}Ap \bigr] \bigr\Vert \leqslant\Delta_{k}, \end{gathered} \end{aligned}$$

where $\nabla f(x_{k})$ is the gradient of $f(x)$ at the current iteration, $H_{f_{k}}$ is either $\nabla^{2} f(x_{k})$ or its approximation. Furthermore, $\Vert \nabla f_{k}^{T}h_{f_{k}} \Vert \leqslant \varepsilon$, where

6 $$ h_{f_{k}}=- \bigl(\nabla f(x_{k})-A^{T} \lambda_{f_{k}} \bigr), $$

and *ε* is a small enough constant, is usually considered as the termination criterion in this class of algorithms.

#### Motivation

The above discussions illustrate that the affine-scaling interior-point trust-region method is an effective way to solve the nonlinear optimization problems with inequality constraints. The trust-region frame guarantees the stable numerical performance. However, in Eqs. (4)–(6) the first- and second-order derivatives play important roles during the computational process, which maybe fail to solve the optimization problems like (1). If both the feasibility and the stability of the algorithm need to be guaranteed, we should consider the derivative-free trust-region methods.

### Derivative-free technique for trust-region subproblem

Since the first- or second-order derivatives of objective functions are not explicitly available, the derivative-free optimization algorithms have been favored by researchers for a time. The application forms of the derivative-free theory are devise [7, 8] and widely applied. Reference [9] proposed a derivative-free algorithm for least-squares minimization, and proved the local convergence in [10]. Reference [11] presented a derivative-free approach to constrained multiobjective nonsmooth optimization. Reference [12] presented a higher-order contingent derivative of perturbation maps in multiobjective optimization. In [13], Conn proposed an unconstrained derivative-free trust-region method. They constructed the trust-region subproblem

$$\begin{aligned} \min_{s\in B(0;\Delta_{k})} m_{k}=m(x_{k}+s)=m(x_{k})+s^{T}g_{k}+ \frac{1}{2}s^{T}H_{m_{k}}s \end{aligned}$$

by using a polynomial interpolation technique, where $\nabla m(x_{k})=g_{k}$, and $\nabla^{2} m(x_{k})=H_{m_{k}}$. Following this idea, we consider that $Y _{k}=\{y_{k}^{0}, y_{k}^{1},\ldots, y_{k}^{t}\}$ is an interpolation sample set around the current iteration point $x_{k}$, and we construct the trust-region subproblem

7 $$ \begin{gathered} \min\quad q_{m_{k}}(p)=g_{k}^{T}p+ \frac{1}{2}p^{T}H_{m_{k}}p+\frac{1}{2}p^{T}A^{T}D_{k}^{-1}C_{m_{k}}Ap \\ \mbox{s.t.}\quad \bigl\Vert \bigl[p;D_{k}^{-{\frac{1}{2}}}Ap \bigr] \bigr\Vert \leqslant\Delta_{k}. \end{gathered} $$

$C_{m_{k}}\stackrel{\mathrm{def}}{=}\operatorname {diag}\{ \vert \lambda _{m_{k}} \vert \}$ with $\lambda_{m_{k}}$ obtained from

8 $$\begin{aligned}& \left [ \begin{matrix} A^{T} \\ -D_{k}^{\frac{1}{2}} \end{matrix} \right ] \lambda_{m_{k}}\stackrel{\mathrm{L.S.}}{=} \left [ \begin{matrix} g_{k} \\ 0 \end{matrix} \right ] , \end{aligned}$$

9 $$\begin{aligned}& h_{m_{k}}=- \bigl(g_{k}-A^{T}\lambda _{m_{k}} \bigr). \end{aligned}$$

We should note that the gradient and Hessian in (5) and (7), (4) and (8), (6) and (9) are different. Meanwhile, since the algorithm in this paper adopts both the decrease direction *p* and the stepsize *α* to update the iteration point, we give a new definition of the error bounds between the objective function $f(x_{k}+\alpha p)$ and the approximation function $m(x_{k}+\alpha p)$ to ensure the global convergence. We shall show the details after assumption (A1).

#### Assumption

1. Suppose that a level set $\mathcal{L}(x_{0})$ and a maximal radius $\Delta_{\max}$ are given. Assume that *f* is twice continuously differentiable with Lipschitz continuous Hessian in an appropriate open domain containing the $\Delta_{\max}$ neighborhood $\bigcup_{x\in\mathcal{L}(x_{0})}B(x, \Delta_{\max})$ of the set $\mathcal {L}(x_{0})$.

#### Definition 1

Given a function *f* satisfies (A1). $\mathcal{M}= \{ m:\Re ^{n}\rightarrow\Re, m \in C^{2} \} $ is a set of model functions. If there exist positive constants $\kappa_{ef}$, $\kappa_{eg}$, $\kappa _{eh}$, and $\kappa_{blh}$, such that, for any $x\in\mathcal {L}(x_{0})$, $\Delta\in(0,\Delta_{\max}]$, and $\alpha\in(0,1]$, there is a model function $m(x+\alpha p) \in\mathcal{M}$, with Lipschitz continuous Hessian and corresponding Lipschitz constant bounded by $\kappa_{blh}$, and such that:

1. the error between the Hessian of the model $m(x +\alpha p)$ and the Hessian of the function $f(x +\alpha p)$ satisfies
  10 $$ \bigl\Vert \nabla^{2}f(x+\alpha p)-\nabla ^{2}m(x+\alpha p) \bigr\Vert \leqslant\kappa_{eh} \alpha \Delta, \quad \forall p \in B(0,\Delta); $$
2. the error between the gradient of the model $m(x +\alpha p)$ and the gradient of the function $f(x +\alpha p)$ satisfies
  11 $$ \bigl\Vert \nabla f(x+\alpha p)-\nabla m(x +\alpha p) \bigr\Vert \leqslant\kappa_{eg} \alpha^{2} \Delta ^{2}, \quad \forall p \in B(0,\Delta); $$
3. the error between the model $m(x +\alpha p)$ and the function $f(x +\alpha p)$ satisfies
  12 $$ \bigl\Vert f(x +\alpha p)- m(x +\alpha p) \bigr\Vert \leqslant \kappa_{ef} \alpha^{3} \Delta^{3}, \quad \forall p \in B(0, \Delta). $$

Such a model *m* is called fully quadratic on $B(x,\Delta)$.

In this paper, we aim to present a class of derivative-free trust-region method for nonlinear programming with linear inequality constraints. The main features of this paper are:

- We use the derivatives of approximation function $m(x_{k}+\alpha p)$ to replace the derivatives of objective function $f(x_{k}+\alpha p)$ to reduce the algorithm’s requirement for gradient and Hessian of the iteration points. We solve an affine-scaling trust-region subproblem to find a feasible search direction in each iteration.
- In the *k*th iteration, a feasible search direction *p* is obtained from an affine-scaling trust-region subproblem. Meanwhile, interior backtracking skill will be applied both for determining stepsize *α* and for guaranteeing the feasibility of iteration point.
- We will show that the iteration points generated by the proposed algorithm could converge to the optimal points of (1).
- Local convergence will be given under some reasonable assumptions.

This paper is organized as follows: we describe a class of derivative-free trust-region method in Sect. 2. The main results including global convergence property and local convergence rate will be discussed in Sect. 3. The numerical results will be illustrated in Sect. 4. Finally, we give some conclusions.

#### Notation

In this paper, $\Vert \cdot \Vert $ is the 2-norm for a vector and the induced 2-norm for a matrix. $B\subset\Re^{n}$ is a closed ball and $B(x,\Delta)$ is the closed ball centered at *x*, with radius $\Delta>0$. *Y* is a sample set and $\mathcal{L}(x_{0})= \{ x\in\Re ^{n}|f(x)\leqslant f(x_{0}),A x\geqslant b \} $ is the level set about the objective function *f*. We use the subscript $f_{k}$ and subscript $m_{k}$ to distinguish the relevant information between the original function and the approximate function. For example, $H_{f_{k}}$ is the Hessian of *f* at *k*th iteration and $H_{m_{k}}$ is the Hessian of $m_{k}$ at *k*th iteration.

## A derivative-free trust region method with interior backtracking technique

To solve the optimization problem (1) with not all available first- or second-order derivatives, we design a derivative-free trust-region method. An affine-scaling matrix is denoted by (3) for linear inequality constraints. We chose a stepsize $\alpha_{k}$ satisfying the following inequalities:

13a $$\begin{aligned} &f(x_{k}+\alpha_{k}p_{k})\leqslant f(x_{k})+\alpha_{k}\kappa_{1} g_{k}^{T}p_{k}, \end{aligned}$$

13b $$\begin{aligned} &\quad \mbox{with } x_{k}+\alpha_{k} p_{k}\in \Omega . \end{aligned}$$

Moreover, set

14 $$ \theta_{k}= \textstyle\begin{cases} 1, & \text{if } x_{k}+\alpha_{k} p_{k} \in \operatorname {int}(\Omega), \\ 1-O( \Vert p_{k} \Vert ^{2}), & \text{otherwise}, \end{cases} $$

where $\theta_{k}\in(\theta_{0},1]$, for some $0<\theta_{0}<1$. The $\theta_{k}$ is to ensure the iterative points generated by the algorithm are strictly interior. Combining with (13a), (13b) and (14), this interior backtracking technique is to guarantee the feasibility of the iterative points. The algorithm possesses the trust-region property and the derivative-free technique is reflected in the trust-region subproblem (7) since the gradient $g_{k}$ and Hessian $H_{m_{k}}$ come from the approximation function, which are different from $\nabla f_{k}$ and $H_{f_{k}}$ in (5), satisfying the error bounds (11) and (12). We adopt $\Vert g_{k}^{T}h_{m_{k}} \Vert $ to be a termination criterion. Now we present the derivative-free trust-region method in detail (see Algorithm 1).

**Algorithm 1:**
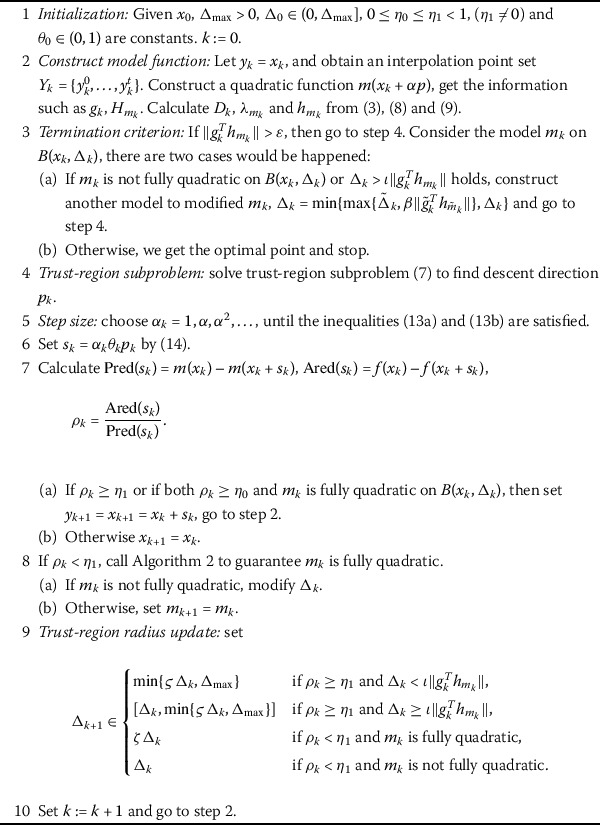
Derivative-free trust-region method with interior backtracking technique

### Remark 1

We add a backtracking interior line-search technique in the algorithm. It is helpful to reducing the number of iterations. Equation (13a) is used to guarantee the descent property of $f(x)$ and (13b) ensures the feasibility of $x_{k}+\alpha_{k} p_{k}$.

### Remark 2

The scalar $\alpha_{k}$, given in step 5, denotes the stepsize along $p_{k}$ to the boundary (13b) of the linear inequality constraints

15 $$ \Gamma_{k}\stackrel{\mathrm{def}}{=}\min \biggl\{ - \frac{a_{i}^{T}x_{k}-b_{i}}{a_{i}^{T}p_{k}}\Big|-\frac {a_{i}^{T}x_{k}-b_{i}}{a_{i}^{T}p_{k}}>0, i=1,2,\ldots,m \biggr\} , $$

with $\Gamma_{k}\stackrel{\mathrm{def}}{=}+\infty$ if $-(a_{i}^{T}x_{k}-b_{i})/(a_{i}^{T}p_{k})\leqslant0$ for all $i=1,2,\ldots,m$. A key property of the scalar $\alpha_{k}$ is that an arbitrary step $\alpha_{k} p_{k}$ to the point $x_{k}+\alpha_{k} p_{k}$ does not violate any linear inequality constraints.

### Remark 3

Let

16 $$ M_{m_{k}}=\left [ \begin{matrix} H_{m_{k}} & 0\\ 0 & C_{m_{k}} \end{matrix} \right ] . $$

The first-order necessary conditions of (7) implies that there exists $v_{m_{k}} \geqslant0$ such that

17 $$ \begin{aligned} &(M_{m_{k}}+v_{m_{k}}I) \left [ \begin{matrix}p_{k} \\ \hat{p}_{k} \end{matrix} \right ] =-\left [ \begin{matrix}g_{k}\\ 0 \end{matrix} \right ] +\left [ \begin{matrix} A^{T} \\ -D_{k}^{\frac{1}{2}} \end{matrix} \right ] \lambda_{m_{k+1}}, \quad \mbox{with } \\ &v_{m_{k}}\left ( \Delta_{k}-\left \Vert \left [ \begin{matrix}p_{k} \\ \hat{p}_{k} \end{matrix} \right ] \right \Vert \right ) =0. \end{aligned} $$

In order to obtain a suitable approximation function, Algorithm 1 needs to update the objective function of the trust-region subproblem if necessary. The model-improvement algorithm is applied only if $\Vert g_{k}^{T}h_{m_{k}} \Vert \leqslant\varepsilon$ and at least one of the following holds: The model $m(x_{k}+ \alpha p)$ is not certifiably fully quadratic on $B(x_{k},\Delta_{k} )$ or $\Delta_{k} > \iota \Vert g_{k}^{T}h_{m_{k}} \Vert $. It improves on the current approximate function $m(x_{k}+\alpha p)$ to meet the requirements of the error bounds so that the model function becomes fully quadratic. We display the model-improvement mechanism in Algorithm 2 which has the same principle as Algorithm 2 proposed in [14], with a constant $\omega \in(0, 1)$.

**Algorithm 2:**
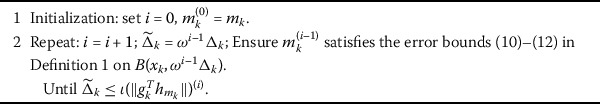
Model-improvement mechanism

## Main results and discussion

In this section, we mainly discuss some properties about the proposed algorithm, including the discussion of the error bounds, the sufficiently descent property, the global and local convergence properties. First of all, we make some necessary assumptions as follows.

### Assumptions

(A2) The level set $\mathcal{L}(x_{0})$ is bounded.
(A3) There exist positive constants $\kappa_{g_{f}}$ and $\kappa_{g_{m}}$ such that $\Vert \nabla{f_{k}} \Vert \leqslant\kappa_{g_{f}}$ and $\Vert g_{k} \Vert \leqslant \kappa_{g_{m}}$, respectively, for all $x_{k}\in\mathcal{L}(x_{0})$.
(A4) There exist positive constants $\kappa_{H_{f}}$ and $\kappa_{H_{g}}$ such that $\Vert H_{f_{k}} \Vert \leqslant \kappa_{H_{f}}$ and $\Vert H_{m_{k}} \Vert \leqslant\kappa _{H_{m}}$, respectively, for all $x_{k}\in\mathcal{L}(x_{0})$.
(A5) $[ \begin{matrix} A & -D_{k}^{\frac{1}{2}} \end{matrix} ] $ is full row rank for all $x_{k}\in\mathcal{L}(x_{0})$.

### Error bounds

Observe first that some error bounds hold immediately.

#### Lemma 1

*Suppose that* (A1)*–*(A5), *the error bounds* (10)*–*(12) *and the fact that* $\Delta_{k}\leqslant\Delta_{\max}$ *hold*. *If* $m_{k}$ *is a fully quadratic model on* $B(x_{k},\Delta_{k})$, *then the following bound is true*:

18 $$\begin{aligned} \Vert h_{f_{k}}-h_{m_{k}} \Vert \leqslant\kappa _{h} \alpha_{k}\Delta_{k}. \end{aligned}$$

#### Proof

Using the theory of matrix perturbation analysis, Eqs. (4) and (8), we obtain

19 $$\begin{aligned} \Vert \lambda_{f_{k}}-\lambda_{m_{k}} \Vert \leqslant \bigl\Vert \bigl(AA^{T}+D_{k} \bigr)^{-1} \bigr\Vert \Vert A \Vert \bigl\Vert \nabla f(x_{k})-g_{k} \bigr\Vert , \end{aligned}$$

where $AA^{T}$ is a positive definition matrix and $D_{k}$ is a diagonal matrix related with $x_{k}\in\mathcal{L}(x_{0})$. By (A2), there exists a constant $\kappa_{\lambda}>0$ such that $\Vert (AA^{T}+D_{k})^{-1} \Vert \Vert A \Vert \leqslant \kappa_{\lambda}$. Thus, from (6), (9) and the error bound (11), one has the fact that

$$\begin{aligned} \Vert h_{f_{k}}-h_{m_{k}} \Vert = & \bigl\Vert \nabla f(x_{k})-g_{k}-A^{T}(\lambda _{f_{k}}-\lambda_{m_{k}}) \bigr\Vert \\ \leqslant& \bigl\Vert \nabla f(x_{k})-g_{k} \bigr\Vert +\kappa_{\lambda} \Vert A \Vert \bigl\Vert \nabla f(x_{k})-g_{k} \bigr\Vert \\ \leqslant& \bigl(1+\kappa_{\lambda} \Vert A \Vert \bigr) \bigl\Vert \nabla f(x_{k})-g_{k} \bigr\Vert \\ \leqslant& \bigl(1+\kappa_{\lambda} \Vert A \Vert \bigr) \kappa_{eg}\alpha_{k}^{2}\Delta _{k}^{2} \\ \leqslant& \bigl(1+\kappa_{\lambda} \Vert A \Vert \bigr) \kappa_{eg}\Delta_{\max}\alpha_{k}\Delta _{k}. \end{aligned}$$

Clearly, the conclusion holds with $\kappa_{h}=(1+\kappa_{\lambda} \Vert A \Vert )\kappa_{eg}\Delta_{\max}$. □

#### Lemma 2

*Suppose that* (A1)*–*(A5), *the error bounds* (10)*–*(12) *and the fact that* $\Delta_{k}\leqslant\Delta_{\max}$ *hold*. *If* $m_{k}$ *is a fully quadratic model on* $B(x_{k},\Delta_{k})$, *for some constant* $\kappa_{2}$, *one has*

20 $$\begin{aligned} \bigl\vert \bigl\Vert \nabla f(x_{k})^{T}h_{f_{k}} \bigr\Vert - \bigl\Vert g_{k}^{T}h_{m_{k}} \bigr\Vert \bigr\vert \leqslant\kappa_{2} \alpha_{k}\Delta _{k}. \end{aligned}$$

#### Proof

Using the triangle inequality, Cauchy–Schwarz inequality, (18), (A3), the error bounds (10)–(12) and the fact that $\alpha_{k}\in(0,1]$ and $\Delta_{k}\leqslant\Delta_{\max}$ successively, we obtain

$$\begin{aligned}& \bigl\vert \bigl\Vert \nabla f(x_{k})^{T}h_{f_{k}} \bigr\Vert - \bigl\Vert g_{k}^{T}h_{m_{k}} \bigr\Vert \bigr\vert \\& \quad \leqslant \bigl\Vert \nabla f(x_{k})-g_{k} \bigr\Vert \Vert h_{f_{k}} \Vert + \Vert g_{k} \Vert \Vert h_{m_{k}}-h_{f_{k}} \Vert \\& \quad \leqslant \bigl\Vert \nabla f(x_{k})-g_{k} \bigr\Vert \Vert h_{f_{k}} \Vert +\kappa_{h} \alpha _{k} \Delta_{k} \Vert g_{k} \Vert \\& \quad \leqslant (\kappa_{eg}\kappa_{g_{f}}\Delta _{\max}+\kappa_{g_{m}}\kappa_{h})\alpha _{k} \Delta_{k}, \end{aligned}$$

which implies that the inequality (20) holds with $\kappa _{2}=\kappa_{eg}\kappa_{g_{f}}\Delta_{\max}+\kappa_{g_{m}}\kappa_{h}$. □

#### Lemma 3

*Suppose that* (A1)*–*(A5), *the error bounds* (10)*–*(12) *and the fact that* $\Delta_{k}\leqslant\Delta_{\max}$ *hold*. *If* $\Vert \nabla f_{k}^{T}h_{f_{k}} \Vert \neq0$, *then step *3 *of Algorithm *1 *will stop in a finite number of improvement steps*.

#### Proof

Now we should prove that $\Vert \nabla f_{k}^{T}h_{f_{k}} \Vert $ must be zero if the loop of Algorithm 2 is infinite.

In fact, there are two cases could cause Algorithm 2 to be implemented. One is that $m_{k}$ is not fully quadratic, the other is that the radius $\Delta_{k}>\iota \Vert g_{k}^{T}h_{m_{k}} \Vert $. Then set $m_{k}^{(0)}=m_{k}$, and improve the model to be fully quadratic on $B(x_{k}, \Delta_{k})$, which denoted by $m^{(1)}_{k}$. If $(g_{k}^{T}h_{m_{k}})^{(1)}$ of $m_{k}^{(1)}$ satisfies the inequality $\iota \Vert (g_{k}^{T}h_{m_{k}})^{(1)} \Vert \geqslant\Delta_{k}$, Algorithm 2 stops with $\widetilde{\Delta}_{k}=\Delta_{k} \leqslant\iota \Vert (g_{k}^{T}h_{m_{k}})^{(1)} \Vert $.

Otherwise, $\iota \Vert (g_{k}^{T}h_{m_{k}})^{(1)} \Vert <\Delta _{k} $ holds. Algorithm 2 will improve the model on $B(x_{k},\omega\Delta_{k})$ and the resulting model is denoted by $m^{(2)}_{k}$. If $m^{(2)}_{k}$ satisfies $\iota \Vert (g_{k}^{T}h_{m_{k}})^{(2)} \Vert \geqslant\omega\Delta_{k}$, the procedure stops. If not, the radius should be multiplied by *ω* and Algorithm 2 will improve the model on $B(x_{k},\omega^{2} \Delta_{k})$, and go on.

The only case for Algorithm 2 to be infinite is if

$$ \iota \bigl\Vert \bigl(g_{k}^{T}h_{m_{k}} \bigr)^{(i)} \bigr\Vert < \omega^{i-1}\Delta_{k} \quad \mbox{for all }i\geqslant1. $$

It implies

$$ \lim_{i\rightarrow+\infty} \bigl\Vert \bigl(g_{k}^{T}h_{m_{k}} \bigr)^{(i)} \bigr\Vert =0. $$

By the bound (20) $\Vert \nabla f_{k}^{T}h_{f_{k}}-(g_{k}^{T}h_{m_{k}})^{(i)} \Vert \leqslant\kappa_{2}\omega^{i-1} \alpha_{k}\Delta_{k} \quad \text{for all } i\geqslant1$, we obtain

$$\begin{aligned} \bigl\Vert \nabla f_{k}^{T}h_{f_{k}} \bigr\Vert \leqslant& \bigl\Vert \nabla f_{k}^{T}h_{f_{k}}- \bigl(g_{k}^{T}h_{m_{k}} \bigr)^{(i)} \bigr\Vert + \bigl\Vert \bigl(g_{k}^{T}h_{m_{k}} \bigr)^{(i)} \bigr\Vert \\ \leqslant& \kappa_{2}\omega^{i-1} \alpha_{k} \Delta_{k}+\frac{\omega^{i-1}}{\iota} \Delta_{k} \\ \leqslant& \kappa_{2}\omega^{i-1}\Delta_{k}+ \frac{\omega^{i-1}}{\iota} \Delta_{k} \\ \leqslant& \biggl(\kappa_{2}+\frac{1}{\iota} \biggr)\omega ^{i-1} \Delta_{k}. \end{aligned}$$

By the choice of $\omega\in(0,1)$ the above inequality means that $\Vert \nabla f_{k} ^{T}h_{f_{k}} \Vert =0$. The conclusion shows us step 3 will stop in a finite number of improvements. □

### Sufficiently descent property

In order to guarantee the global convergence property of the proposed algorithm, it is necessary to show that a sufficiently descent condition is satisfied at the *k*th iteration. We obtained in [6] if step $p_{k}$ is the optimal point of the trust-region subproblem (7), there is a constant $\kappa_{3}>0$ such that

21 $$\begin{aligned} g_{k}^{T}p_{k} \leqslant& - \kappa_{3} \bigl\Vert g_{k}^{T}h_{m_{k}} \bigr\Vert ^{\frac{1}{2}}\min \biggl\{ \Delta_{k}, \frac{ \Vert g_{k}^{T}h_{m_{k}} \Vert ^{\frac{1}{2}}}{ \Vert M_{m_{k}} \Vert } \biggr\} . \end{aligned}$$

#### Lemma 4

*Suppose that* (A1)*–*(A5) *and the error bounds* (10)*–*(12) *hold*. $p_{k}$ *is the solution of the trust*-*region subproblem* (7). *Then there must exist an appropriate* $\alpha_{k}>0$ *which satisfied inequalities* (13a).

#### Proof

We start by considering the maximal step-length along the trust-region subproblem descent direction that preserves sufficient feasibility in the sense of the (13a). Successively using the mean value theorem and (11), we obviously obtain

22 $$\begin{aligned}& f(x_{k})-f(x_{k}+\alpha p_{k}) \\& \quad = -\alpha\nabla f(x_{k})^{T} p_{k} -\frac{1}{2} \alpha^{2} p_{k}^{T} \nabla ^{2} f(\xi_{k}) p_{k} \\& \quad \geqslant -\kappa_{eg}\alpha^{3}\Delta _{k}^{3} -\alpha g_{k}^{T} p_{k} -\frac{1}{2} \alpha^{2} p_{k}^{T} \nabla^{2} f(\xi_{k}) p_{k} \\& \quad = -\alpha\kappa_{1} g_{k}^{T} p_{k} -\kappa_{eg}\alpha^{3}\Delta _{k}^{3}+ \alpha(\kappa_{1}-1) g_{k}^{T} p_{k} -\frac{1}{2} \alpha ^{2} p_{k}^{T}\nabla^{2} f(\xi _{k}) p_{k}, \end{aligned}$$

where $\xi_{k} \in(x_{k},x_{k}+s_{k})$.

There are two cases that may be considered. The first is $p_{k}^{T}\nabla^{2} f(\xi_{k}) p_{k}\leqslant0$. By canceling the last term of Eqs. (22), (21), $\frac{ \Vert g_{k}^{T}h_{m_{k}} \Vert ^{\frac{1}{2}}}{\kappa _{\lambda_{m}}\kappa_{H_{m}}}\leqslant\Delta_{k}$ for large enough *k* and the fact that $\Delta_{k}\leqslant\Delta _{\max}$, it is thus easy to see that there exists an $\alpha^{*}=[\frac{\kappa_{3}(1-\kappa_{1})\kappa _{H_{m}}\kappa_{\lambda_{m}} }{\kappa_{eg}\Delta_{\max}}]^{\frac {1}{2}}>0$ such that (13a) holds. The second case is $p_{k}^{T}\nabla^{2} f(\xi_{k}) p_{k}>0$. Using the Cauchy–Schwarz inequality and the fact that $\alpha_{k}\in(0,1]$ and $\Delta_{k}\leqslant \Delta_{\max}$, we deduce that

$$\begin{aligned}& f(x_{k})-f(x_{k}+\alpha p_{k})+\alpha\kappa _{1} g_{k}^{T} p_{k} \\& \quad \geqslant -\kappa_{eg}\alpha^{3}\Delta _{k}^{3}+ \alpha(\kappa_{1}-1) g_{k}^{T} p_{k} -\frac{1}{2} \alpha ^{2} \kappa_{H_{f}} \Vert p_{k} \Vert ^{2} \\& \quad \geqslant -\kappa_{eg}\alpha^{2}\Delta_{\max} \Delta^{2}_{k}+ \alpha(\kappa_{1}-1) g_{k}^{T} p_{k} -\frac{1}{2} \alpha ^{2} \kappa_{H_{f}} \Delta_{k}^{2} \\& \quad = \alpha \biggl[ \biggl(-\kappa_{eg}\Delta_{\max}- \frac{1}{2}\kappa_{H_{f}} \biggr)\Delta_{k}^{2} \alpha+ (\kappa_{1}-1) g_{k}^{T} p_{k} \biggr] \\& \quad \geqslant 0, \end{aligned}$$

when $\alpha^{*}=\frac{\kappa_{3}(1-\kappa_{1}) \kappa _{H_{m}}\kappa_{\lambda_{m}}}{\kappa_{eg}\Delta_{\max}+\frac {1}{2}\kappa_{H_{f}} }>0$. Thus the final conclusion obtained. □

We therefore see that it is reasonable to design line-search step criterion in step 5, which provided us a nonincreasing sequence $\{f(x_{k})\}$.

#### Lemma 5

*Let step* $p_{k}$ *be the solution of the trust*-*region subproblem* (7). *Suppose that* (A1)*–*(A5) *hold*. *Then there exists a positive constant* $\kappa_{4}$ *such that step* $p_{k}$ *satisfies the following sufficiently descent condition*:

23 $$ \operatorname {Pred}(s_{k})\geqslant\kappa_{4}\alpha _{k} \theta_{k} \bigl\Vert g_{k}^{T}h_{m_{k}} \bigr\Vert ^{\frac{1}{2}}\min \biggl\{ \Delta_{k}, \frac{ \Vert g_{k}^{T}h_{m_{k}} \Vert ^{\frac{1}{2}}}{ \Vert M_{m_{k}} \Vert } \biggr\} , $$

*for all* $g_{k}$, $h_{m_{k}}$, $\Vert M_{m_{k}} \Vert $, *and* $\Delta_{k}$.

#### Proof

Combining now (7), (17), Lemma 4, $\theta_{k}\in (\theta_{0},1]$ and the fact that $\alpha_{k}\leqslant1$, we get

$$\begin{aligned} \operatorname {Pred}(s_{k}) =&m_{k}-m(x_{k}+s_{k}) \\ =& -\alpha_{k} \theta_{k} g_{k}^{T} p_{k} -\frac{(\alpha_{k} \theta_{k})^{2}}{2} p_{k} ^{T} M_{m_{k}} p_{k} \\ \stackrel{(17)}{=}& -\alpha_{k} \theta _{k} g_{k}^{T} p_{k} + \frac{(\alpha_{k} \theta_{k})^{2}}{2} g_{k}^{T}p_{k}+ \frac{(\alpha_{k} \theta_{k})^{2}}{2}v_{m_{k}} \left \Vert \left [ \begin{matrix} p_{k} \\ \hat{p}_{k} \end{matrix} \right ] \right \Vert ^{2} \\ \geqslant& \alpha_{k}\theta_{k} \biggl( \frac{\alpha_{k}\theta_{k}}{2}-1 \biggr) g_{k}^{T}p_{k} \\ \geqslant& \frac{1}{2}\kappa_{3}\alpha_{k} \theta_{k} \bigl\Vert g_{k}^{T}h_{m_{k}} \bigr\Vert ^{\frac{1}{2}}\min \biggl\{ \Delta_{k}, \frac{ \Vert g_{k}^{T}h_{m_{k}} \Vert ^{\frac{1}{2}}}{ \Vert M_{m_{k}} \Vert } \biggr\} \\ = & \kappa_{4}\alpha_{k}\theta_{k} \bigl\Vert g_{k}^{T}h_{m_{k}} \bigr\Vert ^{\frac{1}{2}} \min \biggl\{ \Delta_{k}, \frac{ \Vert g_{k}^{T}h_{m_{k}} \Vert ^{\frac{1}{2}}}{ \Vert M_{m_{k}} \Vert } \biggr\} . \end{aligned}$$

 □

### Global convergence

Every iteration point in the $k+1$th iteration will be chosen on the region $B(x_{k}, \alpha_{k} \Delta_{k})$. Following the lemma one first shows that the current iteration must be successful if $\alpha_{k} \Delta_{k}$ is small enough.

#### Lemma 6

*Suppose that* (A1)*–*(A5) *and the error bounds* (10)*–*(12) *hold*. $m_{k}$ *is fully quadratic on* $B(x_{k},\Delta_{k})$, $\Vert g_{k}^{T}h_{m_{k}} \Vert \neq0$ *and*

$$\begin{aligned} \alpha_{k}\Delta_{k} \leqslant\Delta_{k} \leqslant\min \biggl\{ \frac{1}{\kappa_{H_{m}}\kappa_{\lambda _{m}}}, \frac{\kappa_{4}(1-\eta_{1})}{2\kappa_{ef}\Delta^{2}_{\max }} \biggr\} \bigl\Vert g_{k}^{T}h_{m_{k}} \bigr\Vert ^{\frac{1}{2}} , \end{aligned}$$

*where* $\kappa_{\lambda_{m}}$ *is the bound of* $C_{m_{k}}$, *for all* $x \in\mathcal{L}(x_{0})$. *Then the* *kth iteration is successful*.

#### Proof

We notice that, for all *k* and the model function $m_{k}$, one has $f(x_{k})=m(x_{k})$. Let $M_{f_{k}}=\bigl[ {\scriptsize\begin{matrix}{} H_{f_{k}} & 0 \cr 0 & C_{f_{k}} \end{matrix}} \bigr] $, from (16) and (A3), we know that $\Vert M_{m_{k}} \Vert \leqslant\kappa_{H_{m}}\kappa_{\lambda_{m}}$. Thus combining $\Delta_{k} \leqslant\frac{ \Vert g_{k}^{T}h_{m_{k}} \Vert ^{\frac{1}{2}}}{\kappa_{H_{m}}\kappa _{\lambda_{m}}}$ with the sufficient decrease condition (23), we immediately get

$$ \operatorname {Pred}(s_{k})\geqslant\kappa_{4}\alpha_{k} \theta_{k} \bigl\Vert g_{k}^{T}h_{m_{k}} \bigr\Vert ^{\frac{1}{2}}\min \biggl\{ \Delta_{k}, \frac{ \Vert g_{k}^{T}h_{m_{k}} \Vert ^{\frac{1}{2}}}{ \Vert M_{m_{k}} \Vert } \biggr\} \geqslant\kappa_{4}\alpha_{k} \theta_{k} \bigl\Vert g_{k}^{T}h_{m_{k}} \bigr\Vert ^{\frac{1}{2}}\Delta_{k}. $$

Using Eqs. (12), (23), the fact that $\alpha_{k}\in (0,1]$ and $\theta_{k}\in(0,1]$, we have

$$\begin{aligned} \vert \rho_{k}-1 \vert =& \biggl\vert \frac {f(x_{k})-f(x_{k+1})}{m(x_{k})-m(x_{k+1})}-1 \biggr\vert \\ \leqslant& \biggl\vert \frac {f(x_{k})-m(x_{k})}{m(x_{k})-m(x_{k+1})} \biggr\vert + \biggl\vert \frac{m(x_{k+1})-f(x_{k+1})}{m(x_{k})-m(x_{k+1})} \biggr\vert \\ \stackrel{f(x_{k})=m(x_{k})}{\leqslant} & \frac{2\kappa_{ef}\alpha_{k}^{3}\theta_{k}^{3}\Delta_{k}^{3} }{\kappa_{4}\alpha_{k} \theta_{k} \Vert g_{k}^{T}h_{m_{k}} \Vert ^{\frac{1}{2}}\Delta_{k}} \\ \stackrel{\theta_{k}\leqslant1}{\leqslant} &\frac{2\kappa _{ef}\Delta^{2}_{\max}\alpha_{k}\Delta_{k} }{\kappa_{4} \Vert g_{k}^{T}h_{m_{k}} \Vert ^{\frac{1}{2}}} \\ \leqslant&1-\eta_{1}. \end{aligned}$$

Thus $\rho_{k} \geqslant\eta_{1}$ and the iteration is successful. □

#### Lemma 7

*Suppose that* (A1)*–*(A5) *and the error bounds* (10)*–*(12) *hold*. *If the number of successful iteration is finite*, *then*

$$ \lim_{k\rightarrow+\infty} \bigl\Vert \nabla f(x_{k})^{T}h_{f_{k}} \bigr\Vert = 0. $$

#### Proof

We consider that all the model-improving iterations before $m_{k}$ becomes fully quadratic are less than a constant *N*. Suppose that the current iteration is an iteration after a successful one. It means that an infinite number of iterations are acceptable or not nice. In these two cases, $\Delta_{k}$ is shrinking. Furthermore, $\Delta_{k}$ is reduced by a factor *ζ* at least once every *N* iterations, which implies $\Delta_{k}\rightarrow0$.

For the *j*th iteration, we denote the *i*th iteration after *j* by the index $i_{j}$, then

$$ \Vert x_{j} - x_{i_{j}} \Vert \leqslant N\Delta _{j} \rightarrow0, \quad j\rightarrow+\infty. $$

Using the triangle inequality, we obtain

$$\begin{aligned} \bigl\Vert \nabla f(x_{j})^{T}h_{f_{j}} \bigr\Vert \leqslant& \bigl\Vert \nabla f(x_{j})^{T}h_{f_{j}}- \nabla f(x_{j})^{T}h_{f_{i_{j}}} \bigr\Vert + \bigl\Vert \nabla f(x_{j})^{T}h_{f_{i_{j}}}+ \nabla f(x_{i_{j}})^{T}h_{f_{i_{j}}} \bigr\Vert \\ & {} + \bigl\Vert \nabla f(x_{i_{j}})^{T}h_{f_{i_{j}}}-g_{i_{j}}^{T}h_{f_{i_{j}}} \bigr\Vert + \bigl\Vert g_{i_{j}}^{T}h_{f_{i_{j}}}-g_{i_{j}}^{T}h_{m_{i_{j}}} \bigr\Vert + \bigl\Vert g_{i_{j}}^{T}h_{m_{i_{j}}} \bigr\Vert . \end{aligned}$$

The following work is to show that all these terms on the right-hand side are converging to zero. Because of the Lipschitz continuity of ∇*f* and the fact that $\Vert x_{i_{j}} -x_{j} \Vert \rightarrow0$ the first and second terms converge to zero. The inequalities (10) and (11) imply the third and fourth terms on the right-hand side are converging to zero. According to Lemma 3, if $\Vert g_{i_{j}}^{T}h_{m_{i_{j}}} \Vert \nrightarrow0$ for small enough $\Delta_{i_{j}}$, $i_{j}$ would be a successful iteration, which yield a contradiction. Thus the last term converges to zero. □

#### Lemma 8

*Suppose that* (A1)*–*(A5), *the error bounds* (10)*–*(12) *and* (23) *hold*. *Suppose furthermore that the strict complementarity of the problem* (1) *holds*. *Then*

$$ \liminf_{k\rightarrow+\infty} \bigl\Vert g_{k}^{T}h_{m_{k}} \bigr\Vert = 0. $$

#### Proof

The key is that we may find a contradiction with the fact that $\{f(x_{k})\}$ is a nonincreasing bounded sequence unless $x_{k}$ is a stationary point. We thus have to verify that there exists some $\epsilon>0$ such that $\{f(x_{k})\}$ is not convergent under the assumption of $\Vert g_{k}^{T}h_{m_{k}} \Vert \geqslant\epsilon^{2}$. We observe from (13a), Lemma 4 and (21) that

24 $$\begin{aligned} f(x_{k})-f(x_{k}+\alpha _{k} p_{k}) \geqslant& -\alpha_{k} \kappa _{1} g_{k}^{T}p_{k} \\ \geqslant& \alpha_{k} \kappa_{1} \kappa _{3} \bigl\Vert g_{k}^{T}h_{m_{k}} \bigr\Vert ^{\frac{1}{2}}\min \biggl\{ \Delta_{k}, \frac{ \Vert g_{k}^{T}h_{m_{k}} \Vert ^{\frac{1}{2}}}{ \Vert M_{m_{k}} \Vert } \biggr\} \\ \geqslant& \alpha_{k} \kappa_{1} \kappa _{3} \epsilon\min \biggl\{ \Delta_{k}, \frac{\epsilon}{ \Vert M_{m_{k}} \Vert } \biggr\} \rightarrow0. \end{aligned}$$

Thus from (24), two cases should be considered next, that is,

25 $$\begin{aligned} \liminf_{k\rightarrow\infty}\alpha_{k}\neq0 \end{aligned}$$

and

26 $$\begin{aligned} \lim_{k\rightarrow\infty}\Delta_{k} \neq0 . \end{aligned}$$

We now start the proof of (25). On one hand, $\alpha_{k}$ is accepted by (13b) the boundary of inequality constraints along $p_{k}$. From Eq. (15)

$$ \Gamma_{k}=\min \biggl\{ -\frac {a_{i}^{T}x_{k}-b_{i}}{a_{i}^{T}p_{k}}\Big|-\frac {a_{i}^{T}x_{k}-b_{i}}{a_{i}^{T}p_{k}}>0, i=1,2,\ldots,m \biggr\} , $$

with $\alpha_{k}=+\infty$ if $-(a_{i}^{T}x_{k}-b_{i})/(a_{i}^{T}p_{k})\leqslant0$ for all $i=1,2,\ldots,m$, $\hat{p}_{k}=D_{k}^{-\frac{1}{2}}Ap_{k}$ and (17), we know that there exists $\lambda_{m_{k+1}}$ such that

$$ a_{i}^{T}p_{k}= \bigl(a_{i}^{T}p_{k}-b_{i} \bigr)^{\frac{1}{2}}\hat{p}_{k}^{i} =-\frac {(a_{i}^{T}p_{k}-b_{i})\lambda^{i}_{m_{k+1}}}{v_{m_{k}}+ \vert \lambda^{i}_{m_{k+1}} \vert }, $$

where $\hat{p}_{k}^{i}$ and $\lambda^{i}_{m_{k+1}}$ are the *i*th components of the vectors $\hat{p}_{k}$ and $\lambda_{m_{k+1}}$, respectively. Hence, there exists $j\in{1,\ldots,m}$ such that

27 $$\begin{aligned} \alpha_{k}=-\frac {a_{j}^{T}x_{k}-b_{j}}{a_{j}^{T}p_{k}}\geqslant \frac{v_{m_{k}}+ \vert \lambda^{j}_{m_{k+1}} \vert }{\lambda ^{j}_{m_{k+1}}} \geqslant\frac{v_{m_{k}}+ \vert \lambda ^{j}_{m_{k+1}} \vert }{ \Vert \lambda_{m_{k+1}} \Vert _{\infty}}. \end{aligned}$$

From (17), we have

$$ \left [ \begin{matrix} A^{T} \\ -D_{k}^{\frac{1}{2}} \end{matrix} \right ] \lambda _{m_{k+1}} =-\left [ \begin{matrix}g_{k}\\ 0 \end{matrix} \right ] +(M_{m_{k}}+v_{m_{k}}I) \left [ \begin{matrix} p_{k} \\ \hat{p}_{k} \end{matrix} \right ] . $$

Since $[ \begin{matrix} A & -D_{k}^{\frac{1}{2}} \end{matrix} ] ^{T}$ is full row rank for all $x\in\mathcal{L}(x_{0})$, ${\lambda_{m_{k}}}$ is bounded and $m(x)$ is twice continuously differentiable, there exist $\kappa _{5}>0$ and $\kappa_{6}>0$ such that

$$ \Vert \lambda_{m_{k+1}} \Vert _{\infty}\leqslant\kappa _{5}+(\kappa_{6}+v_{m_{k}})\Delta_{k}. $$

Using the fact that $v_{m_{k}}( \Delta_{k}-\Vert \bigl( {\scriptsize\begin{matrix}{} p_{k} \cr \hat{p}_{k} \end{matrix}} \bigr) \Vert ) =0$ and taking the norm to both sides of (17), we deduce that

$$\begin{aligned} v_{m_{k}}\Delta_{k} =& v_{m_{k}}\left \Vert \left ( \begin{matrix} p_{k}\\ \hat{p}_{k} \end{matrix} \right ) \right \Vert \\ \geqslant& \bigl( \bigl\Vert g_{k}-A^{T}\lambda _{m_{k}} \bigr\Vert ^{2} + \bigl\Vert D_{k}^{\frac{1}{2}} \lambda_{m_{k}} \bigr\Vert ^{2} \bigr)^{\frac{1}{2}}- \Vert M_{m_{k}} \Vert \bigl\Vert (p_{k};\hat{p}_{k}) \bigr\Vert \\ =& \bigl\Vert g_{k}^{T}h_{m_{k}} \bigr\Vert ^{\frac{1}{2}}- \Vert M_{m_{k}} \Vert \bigl\Vert (p_{k}; \hat{p}_{k}) \bigr\Vert . \end{aligned}$$

And noting $\Vert (p_{k};\hat{p}_{k}) \Vert \leqslant\Delta_{k}$, we can obtain

$$ v_{m_{k}}\geqslant\frac{ \Vert g_{k}^{T}h_{m_{k}} \Vert ^{\frac{1}{2}}}{\Delta_{k}}- \Vert M_{m_{k}} \Vert . $$

Combining the assumption $\Vert g_{k}^{T}h_{m_{k}} \Vert >\epsilon^{2}$ with $\Delta_{k}\rightarrow0$ deduced from (24), it is clear from the fact $\Vert M_{m_{k}} \Vert \leqslant\kappa_{\lambda _{m}}\kappa_{H_{m}}$ that, for ∀*k*,

$$ \lim_{k \rightarrow\infty}v_{m_{k}}=+\infty. $$

Thus (27) implies that

$$ \lim_{k \rightarrow\infty}\alpha_{k}\neq0. $$

Furthermore, $\Delta_{k}\rightarrow0$ means that $\lim_{k\rightarrow \infty} \Vert p_{k} \Vert =0$, from which we deduce that, for some $0< \theta_{0} <1 $ and $\theta_{k}-1=O( \Vert p_{k} \Vert ^{2})$, the strictly feasible stepsize $\theta_{k} \in(\theta _{0},1]\rightarrow1$. From the above, we have already seen that (25) holds in the case that $\alpha_{k}$ is determined by (13b).

There is another case that $\alpha_{k}$ is determined by (13a). In this case, we are able to verify that $\alpha _{k}=1$ is acceptable when *k* sufficiently large. If not,

$$\begin{aligned} \kappa_{1}g_{k}^{T}p_{k}< f(x_{k}+p_{k} )-f(x_{k}) \end{aligned}$$

must hold. Applying the Taylor series, (10)–(11), (A3) and the fact that $\Delta_{k}\leqslant\Delta_{\max}$, we deduce that

$$\begin{aligned} \kappa_{1}g_{k}^{T}p_{k} < &f(x_{k}+p_{k})-f(x_{k})= \nabla f(x_{k})^{T}p_{k}+\frac{1}{2}p_{k}^{T} \nabla^{2} f(\xi_{k})p_{k} \\ \leqslant& \biggl(\kappa_{eg}\Delta_{\max}+ \frac{1}{2}\kappa_{eh}\Delta_{\max}+\frac{1}{2} \kappa_{H_{m}} \biggr)\Delta_{k}^{2} +g_{k}^{T}p_{k}, \end{aligned}$$

where $\xi_{k} \in(x_{k},x_{k}+s_{k})$. This inequality is equivalent to the form of

$$\begin{aligned} (1-\kappa_{1})g_{k}^{T}p_{k}+ \biggl( \kappa_{eg}\Delta_{\max}+\frac{1}{2}\kappa _{eh}\Delta_{\max}+\frac{1}{2}\kappa_{H_{m}} \biggr)\Delta_{k}^{2}>0. \end{aligned}$$

Moreover, (21) and $\Vert g_{k}^{T}h_{m_{k}} \Vert \geqslant\epsilon^{2}$ imply that

$$\begin{aligned} -(1-\kappa_{1})\kappa_{3}\epsilon\min \biggl\{ \Delta _{k},\frac{\epsilon}{\kappa_{H_{m}}} \biggr\} + \biggl(\kappa _{eg}\Delta _{\max}+\frac{1}{2}\kappa_{eh}\Delta_{\max}+ \frac{1}{2}\kappa_{H_{m}} \biggr)\Delta_{k}^{2} >0. \end{aligned}$$

Thus if $\Delta_{k}\leqslant\frac{2(1-\kappa_{1})\kappa_{3}\epsilon }{2\kappa_{eg}\Delta_{\max}+\kappa_{eh}\Delta_{\max}+\kappa_{H_{m}}} \leqslant\frac{\epsilon}{\kappa_{H_{m}}}$ we deduce from the inequality

$$\begin{aligned} \Delta_{k} \biggl[ \Delta_{k} \biggl(\kappa _{eg}\Delta_{\max}+ \frac{1}{2}\kappa_{eh} \Delta_{\max}+\frac{1}{2}\kappa_{H_{m}} \biggr) -(1-\kappa _{1})\kappa_{3}\epsilon \biggr] > 0 \end{aligned}$$

that

$$\begin{aligned} \Delta_{k}>\frac{2(1-\kappa_{1})\kappa_{3}\epsilon}{2\kappa _{eg}\Delta_{\max}+\kappa_{eh}\Delta_{\max}+\kappa_{H_{m}}}. \end{aligned}$$

Clearly, a contradiction appears here. It implies that $\alpha_{k}=1$ for *k* sufficiently large. Therefore (25) always holds.

On the other hand, we should prove that (26) is true. From step 3 of Algorithm 1, we know that

$$ \Delta_{k} \geqslant\iota \bigl\Vert g_{k}^{T}h_{m_{k}} \bigr\Vert . $$

By the assumption that $\Vert g_{k}^{T}h_{m_{k}} \Vert \geqslant\epsilon^{2}$, we obtain

$$ \Delta_{k} \geqslant\iota\epsilon. $$

Whenever $\Delta_{k}$ falls below a constant $\bar{\kappa}_{7}$ given by

$$ \bar{\kappa}_{7} =\min \biggl\{ \frac{\epsilon}{\kappa_{H_{m}}\kappa _{\lambda_{m}}}, \frac{\kappa_{4}\epsilon(1-\eta_{1})}{2\kappa_{ef}\Delta^{2}_{\max }} \biggr\} , $$

the *k*th iteration is either successful or model-improving, and hence from step 9, we are able to deduce both that $\Delta_{k+1} \geqslant \Delta_{k}$ and $\Delta_{k+1} \geqslant\zeta\Delta_{k}$. Combining with the rules of step 9 we conclude that $\Delta_{k+1} \geqslant \min \{ \iota\epsilon,\zeta\bar{\kappa}_{7} \} =\kappa_{7}$. It means that $\Delta_{k}\nrightarrow0$, if $\Vert g_{k}^{T}h_{m_{k}} \Vert \geqslant\epsilon^{2}$.

In conclusion, the sequence $\{f(x_{k})\}$ is not convergent if we suppose that $\Vert g_{k}^{T}h_{m_{k}} \Vert \geqslant\epsilon ^{2}$, which contradicts the fact that $\{f(x_{k})\}$ is a nonincreasing bounded sequence. It implies that

$$ \liminf_{k\rightarrow+\infty} \bigl\Vert g_{k}^{T}h_{m_{k}} \bigr\Vert = 0. $$

 □

#### Lemma 9

*For any subsequence* $\{k_{i}\}$ *such that*

28 $$ \lim_{i \rightarrow+\infty} \bigl\Vert g_{k_{i}}^{T}h_{m_{k_{i}}} \bigr\Vert = 0, $$

*we also have*

29 $$\begin{aligned} \lim_{i \rightarrow+\infty} \bigl\Vert \nabla f_{k_{i}}^{T}h_{f_{k_{i}}} \bigr\Vert = 0. \end{aligned}$$

#### Proof

First, we note that, by (28), $\Vert g_{k_{i}}^{T}h_{m_{k_{i}}} \Vert \leqslant\varepsilon$ when *i* sufficiently large. Thus the criticality step ensures that the model $m_{k_{i}}$ is a fully quadratic function on the ball $B(x_{k_{i}}, \Delta_{k_{i}} )$, with $\Delta _{k_{i}}\leqslant\iota \Vert g_{k_{i}}^{T}h_{m_{k_{i}}} \Vert $ for all *i* sufficiently large (if $\Vert \nabla f_{k_{i}}^{T}h_{f_{k_{i}}} \Vert \neq0$). Then, using the bound (20) on the error between the terminal conditions of function and model, we have

$$ \bigl\Vert \nabla f_{k_{i}}^{T}h_{f_{k_{i}}}-g_{k_{i}}^{T}h_{m_{k_{i}}} \bigr\Vert \leqslant\kappa_{2} \alpha_{k_{i}}\Delta _{k_{i}} \leqslant\kappa_{2} \iota\alpha_{k_{i}} \bigl\Vert g_{k_{i}}^{T}h_{m_{k_{i}}} \bigr\Vert \leqslant \kappa_{2} \iota \bigl\Vert g_{k_{i}}^{T}h_{m_{k_{i}}} \bigr\Vert . $$

As a consequence, we have

$$\begin{aligned} \bigl\Vert \nabla f_{k_{i}}^{T}h_{f_{k_{i}}} \bigr\Vert =& \bigl\Vert \nabla f_{k_{i}}^{T}h_{f_{k_{i}}}-g_{k_{i}}^{T}h_{m_{k_{i}}} \bigr\Vert + \bigl\Vert g_{k_{i}}^{T}h_{m_{k_{i}}} \bigr\Vert \leqslant(\kappa_{2} \alpha_{k_{i}}\iota+1) \bigl\Vert g_{k_{i}}^{T}h_{m_{k_{i}}} \bigr\Vert \\ \leqslant& (\kappa_{2} \iota+1) \bigl\Vert g_{k_{i}}^{T}h_{m_{k_{i}}} \bigr\Vert \end{aligned}$$

for all *i* sufficiently large. But $\Vert g_{k_{i}}^{T}h_{m_{k_{i}}} \Vert \rightarrow0$ implies (29) holds. □

Then we obtain the global convergence derived from Lemmas 8 and 9.

#### Theorem 1

*Suppose that* (A1)*–*(A5), *the error bounds* (10)*–*(12) *and* (23) *hold*. *Suppose furthermore that the strict complementarity of the problem* (1) *holds*. *Let* $\{x_{k}\}\subset\Re^{n}$ *be sequence generated by Algorithm *1. *Then*

$$\begin{aligned} \liminf_{k \rightarrow+\infty} \bigl\Vert \nabla f_{k}^{T}h_{f_{k}} \bigr\Vert =0. \end{aligned}$$

The above theorem shows us there exists a limit point that is first-order critical. In fact, we are able to prove that all limit points of the sequence of iterations are first-order critical.

#### Theorem 2

*Suppose that* (A1)*–*(A5), *the error bounds* (10)*–*(12) *and* (23) *hold*. *Suppose furthermore that the strict complementarity of the problem* (1) *holds*. *Let* $\{x_{k}\}\subset\Re^{n}$ *be sequence generated by Algorithm *1. *Then*

$$\begin{aligned} \lim_{k \rightarrow+\infty} \bigl\Vert \nabla f_{k}^{T}h_{f_{k}} \bigr\Vert =0. \end{aligned}$$

#### Proof

We first obtained from Lemma 7 that the theorem holds in the case when *S* is finite. Hence, we will assume that *S* is infinite. For the purpose of deriving a contradiction, we suppose that there exists a subsequence $\{k_{i}\}$ of successful or acceptable iterations such that

30 $$\begin{aligned} \bigl\Vert \nabla f_{k_{i}}^{T}h_{f_{k_{i}}} \bigr\Vert \geqslant\epsilon^{2}_{1} >0 \end{aligned}$$

for some $\epsilon_{1}>0$ and for all *i*. Then, because of Lemma 9, we obtain

$$\begin{aligned} \bigl\Vert g_{k_{i}}^{T}h_{m_{k_{i}}} \bigr\Vert \geqslant\epsilon^{2}_{2} >0 \end{aligned}$$

for some $\epsilon_{2} >0$ and for all *i* sufficiently large. Without loss of generality, we pick $\epsilon_{2}$ such that

31 $$\begin{aligned} \epsilon_{2}^{2}\leqslant\min \biggl\{ \frac{\epsilon_{1}^{2}}{2(2+\kappa_{eg}\iota)}, \epsilon \biggr\} . \end{aligned}$$

Lemma 8 then ensures the existence, for each $\{k_{i}\}$ in the subsequence, of a first iteration $\ell_{i} > k_{i}$ such that $\Vert g_{\ell_{i}}^{T} h_{m_{\ell_{i}}} \Vert < \epsilon^{2}_{2} $. By removing elements from $\{k_{i}\}$, without loss of generality and without a change of notation, we thus see that there exists another subsequence indexed by $\{\ell_{i}\}$ such that

32 $$\begin{aligned} \bigl\Vert g_{k}^{T}h_{m_{k}} \bigr\Vert \geqslant\epsilon^{2}_{2} \quad \text{for } k_{i} \leqslant k\leqslant\ell_{i} \text{ and } \bigl\Vert g_{\ell_{i}}^{T}h_{m_{\ell_{i}}} \bigr\Vert < \epsilon ^{2}_{2}, \end{aligned}$$

for sufficiently large *i*, with inequality (30) being retained.

We now restrict our attention to the set $\mathcal{K}$ corresponding to the subsequence of iterations whose indices are in the set

$$\begin{aligned} \bigcup_{i\in\mathcal{N}_{0}}\{k\in\mathcal{N}_{0}: k_{i} \leqslant k\leqslant\ell_{i}\}, \end{aligned}$$

where $k_{i}$ and $\ell_{i}$ belong to the two subsequences given above in (31).

We know that $\Vert g_{k}^{T}h_{m_{k}} \Vert \geqslant\epsilon ^{2}_{2} $ for $k\in\mathcal{K}$. From Lemma 8 $\lim_{k\rightarrow+\infty}\alpha_{k} \Delta _{k}=0$ and by Lemma 5 we conclude that for any large enough $k\in\mathcal{K}$ the iteration *k* is either successful if the model is fully quadratic or model-improving otherwise. Moreover, for each $k\in\mathcal{K}\cap S$ we have

$$\begin{aligned} f(x_{k})-f(x_{k}+s_{k}) \geqslant& \eta _{1} \bigl[ m(x_{k})-m(x_{k}+h_{k}) \bigr] \\ \geqslant& \eta_{1} \kappa_{4} \alpha_{k} \theta_{k} \bigl\Vert g_{k}^{T}h_{m_{k}} \bigr\Vert ^{\frac{1}{2}}\min \biggl\{ \Delta_{k}, \frac{ \Vert g_{k}^{T}h_{m_{k}} \Vert ^{\frac{1}{2}}}{ \Vert M_{m_{k}} \Vert } \biggr\} , \end{aligned}$$

and, for any such *k* large enough, $\Delta_{k}\leqslant\frac{\epsilon_{2}}{\kappa_{h_{m}}\kappa _{\lambda_{m}}}$. Hence, we have $\alpha_{k} \theta_{k}\Delta_{k}\leqslant\frac {f(x_{k})-f(x_{k}+s_{k})}{\eta_{1} \kappa_{4}\epsilon_{2}}$ for $k\in\mathcal{K}\cap S$ sufficiently large. Since for any $k\in\mathcal{K}$ large enough the iteration is either successful or model-improving and since for a model-improving iteration $x_{k+1}=x_{k}+s_{k}$, we have, for all *i* sufficiently large,

$$ \Vert x_{k_{i}}-x_{\ell_{i}} \Vert \leqslant\sum ^{\ell_{i}-1}_{\substack{ j=k_{i}\\ j\in\mathcal{K}\cap\mathcal{S}}} \Vert x_{j}-x_{j+1} \Vert \leqslant\sum^{\ell_{i}-1} _{\substack{\scriptstyle j=k_{i}\\ j\in\mathcal{K}\cap\mathcal{S}}}\alpha _{j}\theta_{j}\Delta_{j}\leqslant \frac{1}{\eta_{1} \kappa_{4} \epsilon_{2}} \bigl[f(x_{k_{i}})-f(x_{\ell_{i}}) \bigr]. $$

Because the sequence $\{f(x_{k})\}$ is bounded below and monotonic decreasing, we see that the right-hand side of this inequality must converge to zero, and we therefore obtain

$$ \lim_{i\rightarrow+\infty} \Vert x_{k_{i}}-x_{\ell_{i}} \Vert =0. $$

Now,

$$\begin{aligned} \bigl\Vert \nabla f(x_{k_{i}})^{T}h_{f_{k_{i}}} \bigr\Vert \leqslant& \bigl\Vert \nabla f(x_{k_{i}})^{T}h_{f_{k_{i}}}- \nabla f(x_{\ell_{i}})^{T}h_{f_{\ell_{i}}} \bigr\Vert + \bigl\Vert \nabla f(x_{\ell_{i}})^{T}h_{f_{\ell_{i}}}-g_{\ell _{i}}^{T}h_{m_{\ell_{i}}} \bigr\Vert \\ & {} + \bigl\Vert g_{\ell_{i}}^{T}h_{m_{\ell_{i}}} \bigr\Vert . \end{aligned}$$

Since ∇*f* is Lipschitz continuity, we see that the first term of the above inequality $\Vert \nabla f(x_{k_{i}})^{T}h_{f_{k_{i}}}-\nabla f(x_{\ell_{i}})^{T}h_{f_{\ell _{i}}} \Vert \rightarrow0$ and is bounded by $\epsilon^{2}_{2}$ for *i* sufficiently large. Equation (32) implies the third term $\Vert g_{\ell _{i}}^{T}h_{m_{\ell_{i}}} \Vert \leqslant\epsilon^{2}_{2}$. From (31) we see that $m_{\ell_{i}}$ is a fully quadratic function on $B(x_{\ell_{i}}, \iota \Vert g_{\ell_{i}}^{T}h_{m_{\ell_{i}}} \Vert )$. Using (11) and (32), we deduce that $\Vert \nabla f(x_{\ell_{i}})^{T}h_{f_{\ell_{i}}}-g_{\ell _{i}}^{T}h_{m_{\ell_{i}}} \Vert \leqslant\kappa_{eg} \iota \epsilon^{2}_{2}$ for *i* sufficiently large. Combining with these bounds we obtain the consequence that

$$\begin{aligned} \bigl\Vert \nabla f_{k_{i}}^{T}h_{f_{k_{i}}} \bigr\Vert \leqslant(2+\kappa_{eg}\iota)\epsilon^{2}_{2} \leqslant\frac{1}{2}\epsilon^{2}_{1} \end{aligned}$$

for *i* large enough. This result contradicts (30), which implies the initial assumption is false and the theorem follows. □

### Local convergence

Having proved the global convergence, we now focus on the speed of the local convergence. For this motivation, more acceptable assumptions are given as follows.

#### Assumptions

(A6) $x_{*}$ is the solution of problem (1), which satisfies the strong second-order sufficient condition, that is, let the columns of $Z_{*}$ denote an orthogonal basis for the null space of $[ \begin{matrix} A & -D^{\frac{1}{2}}_{*} \end{matrix} ] $, then there exists $\varpi>0$ such that
  33 $$ d^{T}({Z_{*}}M_{f_{*}}{Z_{*}})d \geqslant\varpi \Vert d \Vert ^{2}, \quad \forall d. $$
(A7) Let
  34 $$ \lim_{k \rightarrow\infty}\frac{ \Vert (M_{m_{k}}-M_{f_{k}})Z_{k} p_{k} \Vert }{ \Vert p_{k} \Vert }=0. $$
  This means that for large *k*
  $$\begin{aligned} p_{k}^{T} \bigl(Z_{k}^{T} M_{m_{k}}Z_{k} \bigr)p_{k} =&p_{k}^{T} \bigl(Z_{k}^{T}M_{f_{k}}Z_{k} \bigr)p_{k}+o \bigl( \Vert p_{k} \Vert ^{2} \bigr). \end{aligned}$$

#### Theorem 3

*Suppose that* (A1)*–*(A7), *the error bounds* (10)*–*(12) *and* (23) *hold*. $\{x_{k}\}$ *is a sequence generated by Algorithm *1. *Suppose furthermore that the strict complementarity of the problem* (1) *holds*. *Then*, *for sufficiently large* *k*, *the stepsize* $\alpha_{k}\equiv1$ *and there exists* $\hat{\Delta}>0$ *such that* $\Delta_{k}\geqslant\Delta_{K'}\geqslant\hat{\Delta}$, $\forall k\geqslant K'$, *where* $K'$ *is a large enough index*.

#### Proof

According to the algorithm, the stepsize $\alpha_{k}$ is given in (15)

$$\begin{aligned} \Gamma_{k}=\min \biggl\{ -\frac{a_{i}^{T}x_{k}-b_{i}}{a_{i}^{T}p_{k}}\Big|- \frac{a_{i}^{T}x_{k}-b_{i}}{a_{i}^{T}p_{k}}>0, i=1,2,\ldots,m \biggr\} . \end{aligned}$$

From $\hat{p}_{k}=D_{k}^{-\frac{1}{2}}Ap_{k}$ and (17), there exists $\lambda_{m_{k+1}}$ such that

35 $$\begin{aligned} a_{i}^{T}p_{k}= \bigl(a_{i}^{T}p_{k}-b_{i} \bigr)^{\frac{1}{2}}\hat{p}_{k}^{i} =-\frac {(a_{i}^{T}p_{k}-b_{i})\lambda^{i}_{m_{k+1}}}{v_{m_{k}}+ \vert \lambda^{i}_{m_{k+1}} \vert }, \end{aligned}$$

where $\hat{p}_{k}^{i}$ and $\lambda^{i}_{m_{k+1}}$ are the *i*th component of the vectors $\hat{p}_{k}$ and $\lambda_{m_{k+1}}$, respectively.

If $\Vert p_{k} \Vert <\Delta_{k}$, then $v_{m_{k}}=0$. Since the strict complementarity of the problem (1) holds at every limit point of $\{x_{k}\}$, i.e., $\vert \lambda _{m_{k+1}}^{T}j \vert + \vert a_{j}^{T}x_{k}-b_{j} \vert >0$, for all large *k*, $\lambda_{m_{k+1}}=\lambda_{m_{k+1}}^{N}>0$ when $v_{m_{k}}=0$. So, $\lambda_{m_{k+1}}^{j}=(\lambda_{m_{k+1}}^{N})^{j}>0$. From (35), it is clear that $\lim_{k \rightarrow\infty}\alpha_{k}=1$.

If $\Vert p_{k} \Vert =\Delta_{k} \rightarrow0$, then $v_{m_{k+1}}\rightarrow\infty$. From (35),

$$\begin{aligned} \alpha_{k}=-\frac{a_{j}^{T}x-b_{j}}{a^{T}_{j} p_{k}}\geqslant\frac {v_{m_{k}}+ \vert \lambda^{j}_{m_{k+1}} \vert }{ \vert \lambda^{j}_{m_{k+1}} \vert } \geqslant \frac{v_{m_{k}}+ \vert \lambda^{j}_{m_{k+1}} \vert }{ \Vert \lambda^{j}_{m_{k+1}} \Vert _{\infty}}\rightarrow\infty. \end{aligned}$$

From the above, we have found that if $\Vert g_{k}^{T}h_{m_{k}} \Vert \geqslant\varepsilon^{2}$ holds and $\Delta_{k}\rightarrow 0$, we conclude that $\lim_{k\rightarrow\infty}\alpha_{k}=+\infty$, and $\lim_{k\rightarrow\infty}\theta_{k}=1$.

Further, by the condition on the strictly feasible stepsize $\theta_{k}-1=O( \Vert p_{k} \Vert )$, and $\lim_{k\rightarrow \infty}p_{k}=0$, we have $\lim_{k\rightarrow\infty}\theta_{k}=1$.

We can obtain from above that $\lim_{k\rightarrow\infty}\Gamma _{k}=+\infty$ when $\alpha_{k}$ is given in (15) along $p_{k}$. It means that if $\alpha_{k}$ is determined by (13b), $\alpha _{k}\equiv1$ for sufficiently large *k*. Thus

36 $$\begin{aligned} f(x_{k}+p_{k}) =& f(x_{k})+\nabla f_{k}^{T}p_{k}+ \frac{1}{2}p_{k}^{T}H_{f_{k}} p_{k}+o \bigl( \Vert p_{k} \Vert ^{2} \bigr) \\ =& f(x_{k})+\kappa_{1}g_{k}^{T}p_{k}+ \biggl(\frac{1}{2}-\kappa_{1} \biggr)g_{k}^{T}p_{k}+ \nabla f_{k}^{T} p_{k}-g_{k}^{T}p_{k} \\ & {} + \frac{1}{2} \bigl(g_{k}^{T}p_{k}+p_{k}^{T}H_{m_{k}} p_{k} \bigr)+\frac{1}{2}p_{k}^{T}(H_{f_{k}}-H_{m_{k}}) p_{k}+o \bigl( \Vert p_{k} \Vert ^{2} \bigr). \end{aligned}$$

The error bound (11) shows us $(g_{k}-\nabla f_{k})^{T} p_{k}=o( \Vert p_{k} \Vert ^{2})$. Hence we see from (36) that $f(x_{k}+p_{k})\leqslant f(x_{k})+\kappa_{1}g_{k}^{T}p_{k} $ at the *k*th iteration.

Combining with the fact that $p_{k}^{T}A^{T}D^{-1}_{k}C_{m_{k}}Ap_{k} \rightarrow0$, we know that $x_{k+1}=x_{k}+p_{k}$. So

$$\begin{aligned}& \bigl\vert f(x_{k})-f(x_{k}+p_{k})-m(x_{k})+m(x_{k}+p_{k}) \bigr\vert \\& \quad = \biggl\vert \biggl[g_{k}^{T}p_{k}+ \frac{1}{2}p_{k}^{T}M_{m_{k}}p_{k} \biggr]- \biggl[\nabla f_{k}^{T}p_{k}+ \frac{1}{2}p_{k}^{T}H_{f_{k}}p_{k}+o \bigl( \Vert p_{k} \Vert ^{2} \bigr) \biggr] \biggr\vert \\& \quad = \biggl\vert (g_{k}-\nabla f_{k})^{T}p_{k}+ \frac{1}{2}p_{k}^{T}(H_{m_{k}}-H_{f_{k}})p_{k}+o \bigl( \Vert p_{k} \Vert ^{2} \bigr) \biggr\vert \\& \quad \stackrel{(10), (11)}{=} o \bigl( \Vert p_{k} \Vert ^{2} \bigr). \end{aligned}$$

By assumptions (A1)–(A7), we can obtain

37 $$\begin{aligned} \rho_{k}-1 =&\frac {f(x_{k})-f(x_{k}+p_{k})+m(x_{k})-m(x_{k}+p_{k})}{\operatorname {Pred}(p_{k})} \\ = &\frac{[g_{k}^{T}p_{k}+\frac{1}{2}p_{k}^{T}M_{m_{k}}p_{k}]-[\nabla f(x_{k})^{T}p_{k}+\frac{1}{2}p_{k}^{T}H_{f_{k}}p_{k}+o( \Vert p_{k} \Vert ^{2})]}{\operatorname {Pred}(p_{k})} \\ =&\frac{o( \Vert p_{k} \Vert ^{2})}{\operatorname {Pred}(p_{k})}. \end{aligned}$$

By (16) and (17), we get

$$\begin{aligned} g_{k}^{T}p_{k} =&\left \{ \left [ \begin{matrix} g_{k} \\ 0 \end{matrix} \right ] +\left [ \begin{matrix} A^{T} \\ -D_{k}^{\frac{1}{2}} \end{matrix} \right ] \lambda_{m_{k+1}} \right \} ^{T}\left [ \begin{matrix} p_{k} \\ \hat{p}_{k} \end{matrix} \right ] \\ =&-\left [ \begin{matrix} p^{T}_{k} , \hat{p}_{k}^{T} \end{matrix} \right ] (M_{m_{k}}+v_{m_{k}}I)\left [ \begin{matrix} p_{k} \\ \hat{p}_{k} \end{matrix} \right ] \\ \leqslant&-\left [ \begin{matrix} p^{T}_{k} , \hat{p}_{k}^{T} \end{matrix} \right ] M_{m_{k}}\left [ \begin{matrix} p_{k} \\ \hat{p}_{k} \end{matrix} \right ] . \end{aligned}$$

Let the columns of $Z_{k}$ denote an orthogonal basis for the null space of $[ \begin{matrix} A & -D_{k}^{\frac{1}{2}} \end{matrix} ] $. We get $g_{k}^{T}p_{k}\leqslant-[ \begin{matrix} p^{T}_{k} , \hat{p}_{k}^{T} \end{matrix} ] M_{m_{k}}\bigl[ {\scriptsize\begin{matrix}{} p_{k} \cr \hat{p}_{k} \end{matrix}} \bigr] =-p^{T}_{k}Z_{k}^{T}M_{m_{k}}Z_{k} p_{k} $. Therefore, from (33)–(34), we see that for all large *k*

$$ g_{k}^{T}p_{k}\leqslant-\frac{\varpi}{2} \Vert p_{k} \Vert ^{2}+o \bigl( \Vert p_{k} \Vert ^{2} \bigr) . $$

Hence, one has

38 $$\begin{aligned} \operatorname {Pred}(p_{k}) =& -g_{k}^{T}p_{k}- \frac{1}{2}p_{k}^{T}M_{m_{k}}p_{k} \\ =& -g_{k}^{T}p_{k}- \frac{1}{2}p_{k}^{T} \bigl(H_{m_{k}}+A^{T}D_{k}^{-1}C_{k}A \bigr)p_{k} \\ =&- g_{k}^{T}p_{k}- \frac{1}{2}p_{k}^{T}M_{f_{k}}p_{k}+o \bigl( \Vert p_{k} \Vert ^{2} \bigr) \\ \geqslant& \frac{\varpi}{4} \Vert p_{k} \Vert ^{2}+o \bigl( \Vert p_{k} \Vert ^{2} \bigr). \end{aligned}$$

For a similar proof, we can obtain $p_{k}\rightarrow0$. Combining (37) with (38), one has the fact that $\rho_{k}\rightarrow1$. Hence there exists $\hat{\Delta}>0$ such that when $\Vert p_{k} \Vert \leqslant\hat{\Delta}$, $\hat{\rho}_{k}\geqslant\rho_{k} \geqslant\eta_{2}$, and $\Delta _{k+1}\geqslant\Delta_{k}$. As $p_{k}\rightarrow0$, there exists an index $K'$ such that $\Vert p_{k} \Vert \leqslant\hat{\Delta}$ whenever $k\geqslant K'$. Thus, the conclusion holds. □

Theorem 3 implies that the local convergence rate of Algorithm 1 depends on the Hessian at $x_{*}$ and the local convergence rate of $p_{k}$. Meanwhile, if $p_{k}$ is a quasi-Newton step, for sufficiently large *k*, the sequence $\{x_{k}\}$ will reach a superlinear local convergence rate to the optimal point $x_{*}$.

## Numerical experiments

We now demonstrate the experiment performance of the proposed derivative-free trust-region method.

*Environment:* The algorithms are written in Matlab R2009a and run on a PC with 2.66 GHz Intel(R) Core(TM)2 Quad CPU and 4 G DDR2.

*Initialization:* The values $\Delta_{0}=2$, $\eta_{0}=0.25$, $\eta_{1}=0.75$, $\zeta=0.5$, $\varsigma=1.5$, $\iota=0.5$, $\beta=0.25$, $\alpha=0.2$, $\varepsilon= 10^{-8}$ and $\omega=0.3$ are used. $\Delta_{\max}$ is equal to 4, 6, 8, respectively.

*Termination criteria:* $\Vert g_{k} ^{T}h_{m_{k}} \Vert \leqslant\varepsilon$.

*Problems:* We first test 20 linear inequality constrained optimization problems (listed in Table 1) from Test Examples for Nonlinear Programming Codes [15, 16]. It is worth noting that the assumptions (A2)–(A5) play very important roles in the theoretical proof. Here (A2) is a general assumption in the optimization problem and (A5) can be satisfied if the iteration points are not optimal. According to the definitions of error bounds in our algorithm, the gradient (or Hessian) of the model function must be bounded if there exists a constant such that the gradient (or Hessian) norm of the objective function is bounded. Therefore, most of the above test problems satisfy the assumptions (A2)–(A5). For example (HS21)

$$\begin{aligned}& \min\quad f(x)=0.01x^{2}_{1}+x_{2}^{2}-100 \\& \text{s.t.}\quad 10x_{1}-x_{2}-10\geqslant0, \\& \hphantom{\text{s.t.}\quad} 2\leqslant x_{1}\leqslant50, \\& \hphantom{\text{s.t.}\quad}-50 \leqslant x_{2}\leqslant50; \\& \bigl\Vert \nabla f(x) \bigr\Vert =\left \Vert \left [ \begin{matrix} 0.02x_{1} \\ 2x_{2} \end{matrix} \right ] \right \Vert \leqslant980.0005, \quad\quad \bigl\Vert \nabla^{2} f(x) \bigr\Vert =\left \Vert \left [ \begin{matrix} 0.02&0 \\ 0&2 \end{matrix} \right ] \right \Vert = 2. \end{aligned}$$

**Table 1:** Test problems

No.	Problem	Dim	$x_{0}$
1	HS21	2	[−1,−1]
3	HS25	3	[100,12.5,3]
5	HS36	3	[10,10,10]
7	HS44	4	[0,0,0,0]
9	HS76	4	[0.5,0.5,0.5,0.5]
11	HS231	2	[−1.2,1]
13	HS224	2	[0.1,0.1]
15	HS250	3	[10,10,10]
17	HS253	3	[0,2,0]
19	HS331	2	[0.5,0.1]
2	HS24	2	[1,0.5]
4	HS35	3	[0.5,0.5,0.5]
6	HS37	3	[10,10,10]
8	HS45	5	[2,2,2,2,2]
10	HS224	2	[0.1,0.1]
12	HS232	2	[2,0.5]
14	HS232	2	[2,0.5]
16	HS251	3	[10,10,10]
18	HS268	5	[1,1,…,1]
20	HS340	3	[1,1,1]

Of course, we will use the level set to limit the bound of $\Vert \nabla f(x) \Vert $ during program execution, which will be much smaller than this value. Even if the boundedness of the gradient and of the Hessian of the objective functions cannot be satisfied at the same time, at least the boundedness within the level set can be guaranteed.

**Figure 1:**
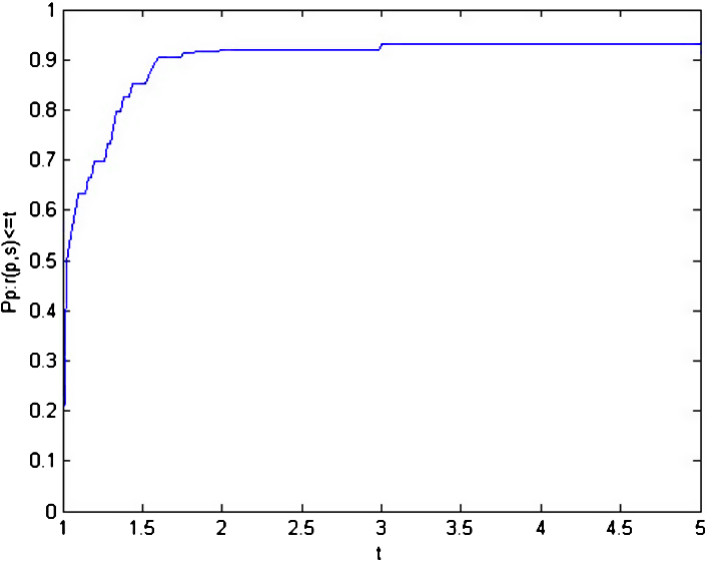
The total iteration number performance of Algorithm 1

**Figure 2:**
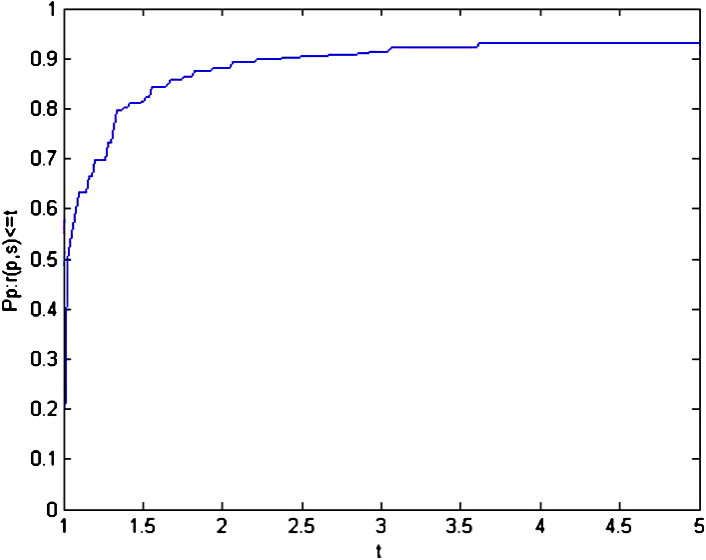
The CPU time performance of Algorithm 1

We use the tool of Dolan and Moré [17] to analyze the efficiency of the given algorithm. Figures 1 and 2 show that Algorithm 1 is feasible and has the robust property.

Furthermore we test five simple linear inequality constrained optimization problems from [16] and compare the experiment results of different trust-region radius upper bound $\Delta_{\max}$. Table 2 shows the experiment results, where *nf* represents the number of function evaluations, *n* is the dimension of the test problems and *F* means the algorithm terminated in the case that the iteration number exceeds the maximum number. The CPU times of the test problems are reported. Table 2 indicates that Algorithm 1 is executable to reach optimal point. The choice of $\Delta_{\max}=6$ is made to enable us to carry out more gratifying results. But the results show that the number of iterations maybe higher than any other derivative-based algorithms. The reason we think is that the derivatives of most of the test problems we chose are available and a derivative-free technique may increase the number of executions; then higher iteration numbers are necessary.

**Table 2:** Experiment results on linear inequality constrained optimization problems

Problem name		Results					
		$\Delta_{\max}=4$		$\Delta_{\max}=6$		$\Delta_{\max}=8$	
	*n*	*nf*	CPUt	*nf*	CPUt	*nf*	CPUt
HS224	2	26	5.187	23	3.35	23	3.35
HS231	2	16	2.025	18	4.018	F	F
HS232	2	8	2.387	23	2.455	F	F
HS250	3	12	55	17	73	16	61
HS251	3	35	3.036	32	2.022	37	2.332

## Conclusions

In this paper, we propose an affine-scaling derivative-free method for linear inequality constrained optimizations.

1. This algorithm is mainly designed to solve the unavailable derivatives optimization problems in engineering. The proposed algorithm adopts interior backtracking technique and possesses the trust-region property.
2. The global convergence is proved by using the definition of fully quadratic. It shows that the iteration points generated by the proposed algorithm could converge to the optimal points of (1). Meanwhile, we get the result that the local convergence rate of the proposed algorithm depends on $p_{k}$. If $p_{k}$ becomes the quasi-Newton step, then the sequence $x_{k}$ generated by the algorithm converges to $x_{*}$ superlinearly.
3. The preliminary numerical experiments verify the new algorithm we proposed is feasible and effective for solving unavailable-derivative linear inequality constrained optimization problems.
